# Surgical resection of mediastinal metastasis from small cell carcinoma of bladder: case report

**DOI:** 10.1186/s44215-022-00003-4

**Published:** 2022-10-04

**Authors:** Tomomi Isono, Toru Kimura, Kenji Kimura, Ryusuke Karube, Koshiro Ando, Hiroto Ishida, Akihiro Nagoya, Seiji Taniguchi, Soichiro Funaki, Yasushi Shintani

**Affiliations:** 1 Department of General Thoracic Surgery, Osaka University Graduate School of Medicine, 2-2 (L5), Yamadaoka, Suita-city, Osaka 565-0871 Japan; 2 Department of Surgery, Toyonaka Municipal Hospital, 4-14-1, Shibaharacho, Toyonaka-city, Osaka 560-8565 Japan; 3 Department of General Thoracic Surgery, Osaka Habikino Medical Center, 3-7-1, Habikino, Habikino-city, Osaka 583-8588 Japan

**Keywords:** Bladder cancer, Mediastinal tumor, Metastasis/metastasectomy, Small cell carcinoma, Thoracotomy

## Abstract

**Background:**

Isolated mediastinal metastasis from a malignant tumor and small cell carcinoma of the bladder are both very rare.

**Case presentation:**

A 76-year-old woman who had undergone surgery for bladder cancer twice was referred to our hospital for a right paracardiac mass noted in chest computed tomography findings, and resection of the tumor was performed. Histological analysis of the mediastinal tumor revealed it to be a metastatic small cell carcinoma of the bladder. At 4 months after surgery, multiple metastatic lesions were found in the chest and liver, and chemotherapy for small cell carcinoma was started.

**Conclusions:**

We present this case of mediastinal metastasis of small cell carcinoma of the bladder, which is very rare, to show the importance of surgical resection of an isolated mediastinal tumor. Such a procedure should be considered, as histological diagnosis of the tumor could be useful for determining therapeutic options.

## Background

Isolated mediastinal metastasis of a malignant tumor [1] and a small cell carcinoma of the bladder [2] are both rare entities. Here, we present findings of a case of metastasis from a small cell carcinoma of the bladder that resulted in a right paracardiac mass showing that surgical resection of an isolated mediastinal tumor could influence the clinical course of the patient.

## Case presentation

**Fig. 1:**
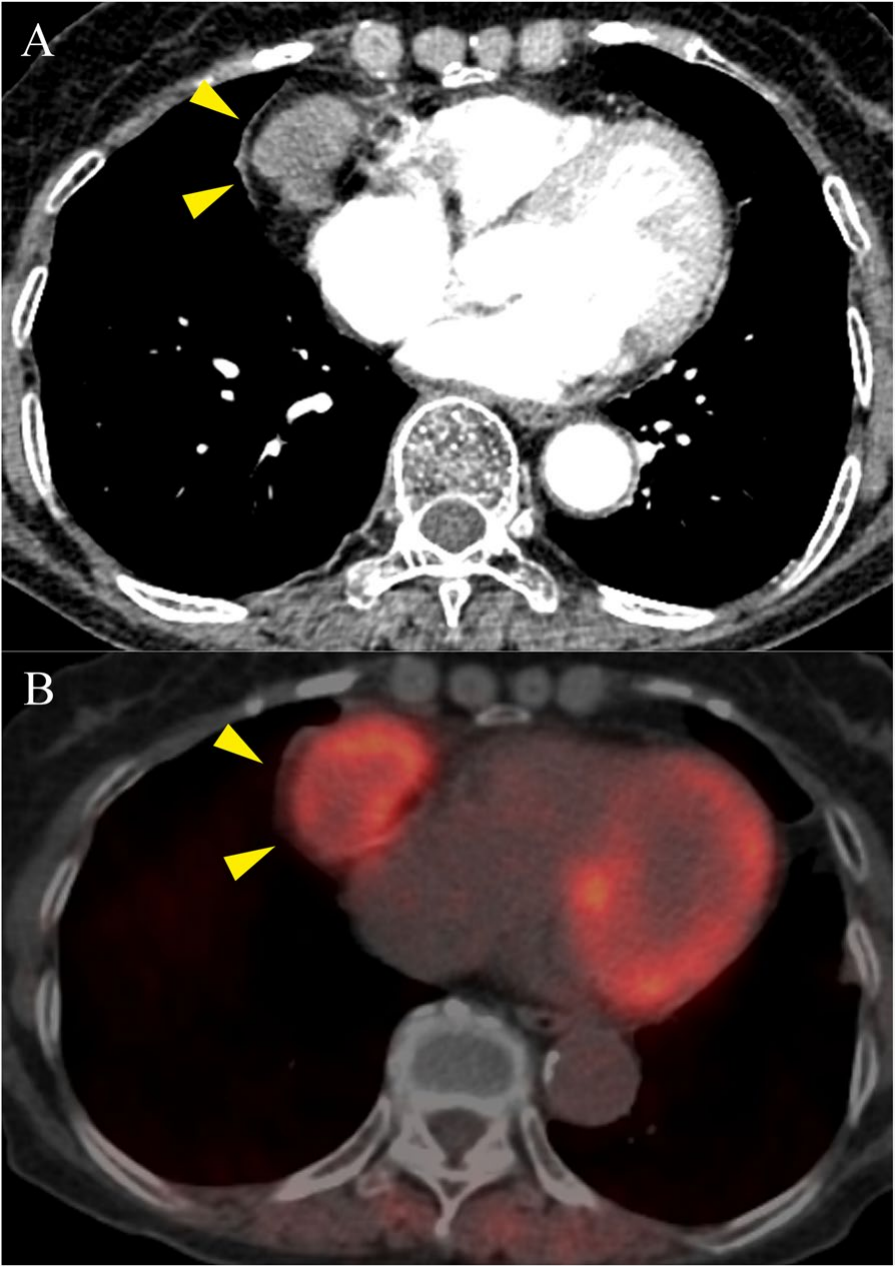
**A** Chest contrast-enhanced computed tomography image showing an enhanced heterogeneous mass, sized 4.5 cm, in the mediastinum. **B** FDG positron emission tomography revealed abnormal FDG uptake (SUVmax = 6.31) in the mass. Yellow triangles in **A** and **B** indicate the tumor

A 76-year-old woman was referred to our hospital for a right paracardiac mass noted in chest contrast-enhanced computed tomography (CT) findings. She had undergone transurethral resection of bladder cancer (BC) 3 years prior and a total cystectomy with ileal conduit diversion for BC recurrence 1 year prior to the referral. The diagnosis for BC was invasive urothelial carcinoma, stage II. Chest CT follow-up examinations as part of the postoperative course of BC were performed every 4 months, which 1 year after the most recent surgery revealed an enhanced heterogeneous mass sized 4.5 cm in the mediastinum (Fig. 1A) with rapid growth features. The serum level of progastrin-releasing peptide, determined for ruling out primary lung cancer, was 257.0 pg/mL (normal < 81.0 pg/mL), though other tumor markers were within normal limits. F-18 fluorodeoxyglucose (FDG) positron emission tomography (PET) showed abnormal FDG uptake in the mass (SUVmax = 6.31) (Fig. 1B), while no other abnormal uptake was detected. Based on these findings, a malignant thymic tumor or BC metastasis was suspected, and we decided to surgically remove the tumor for diagnosis and determining treatment.

**Fig. 2:**
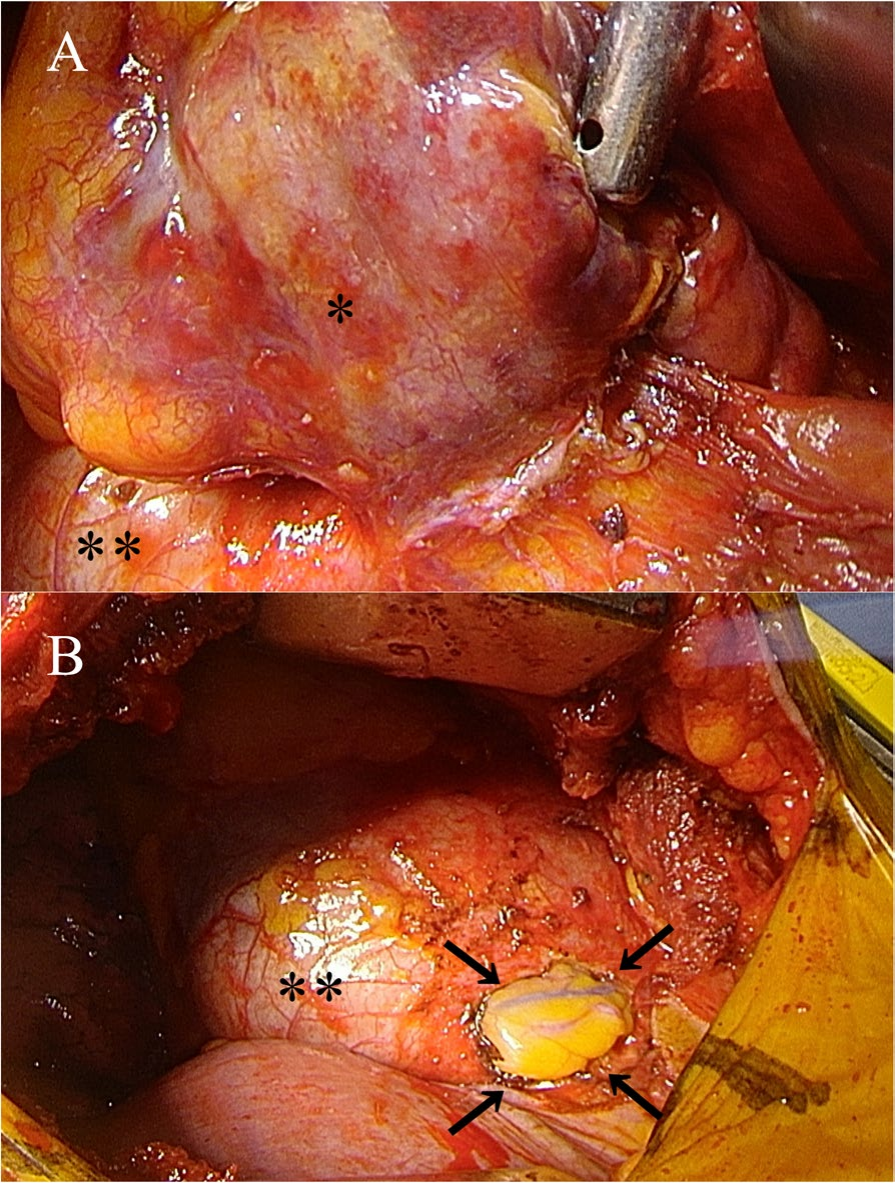
Intraoperative findings. **A** Tumor invasion of pericardium. Single asterisk (*) indicates tumor; double asterisks (**) indicate heart with pericardium. **B** Using a pericardial resection procedure, the tumor was completely resected. Black arrows indicate the pericardium resection line

The tumor was approached via an open thoracotomy at the sixth intercostal space and found to be located in the lower right corner of the anterior mediastinum. The mass was completely surrounded by fatty tissue, and it was difficult to determine whether the fat surrounding the tumor was involuted thymus or pericardial fat. Tumor invasion of the pericardium was noted (Fig. 2A), though there were no tumor cells in the cardiac sac. Resection of the tumor, including the pericardium where the tumor had invaded, was performed (Fig. 2B). Intraoperative rapid diagnosis procedures revealed the resected tumor to be an epithelial malignant tumor, while a more detailed diagnosis could not be determined. No evidence of tumor cells in the resection margin was confirmed. The operation time was 122 min, and blood loss was 200 ml.

**Fig. 3:**
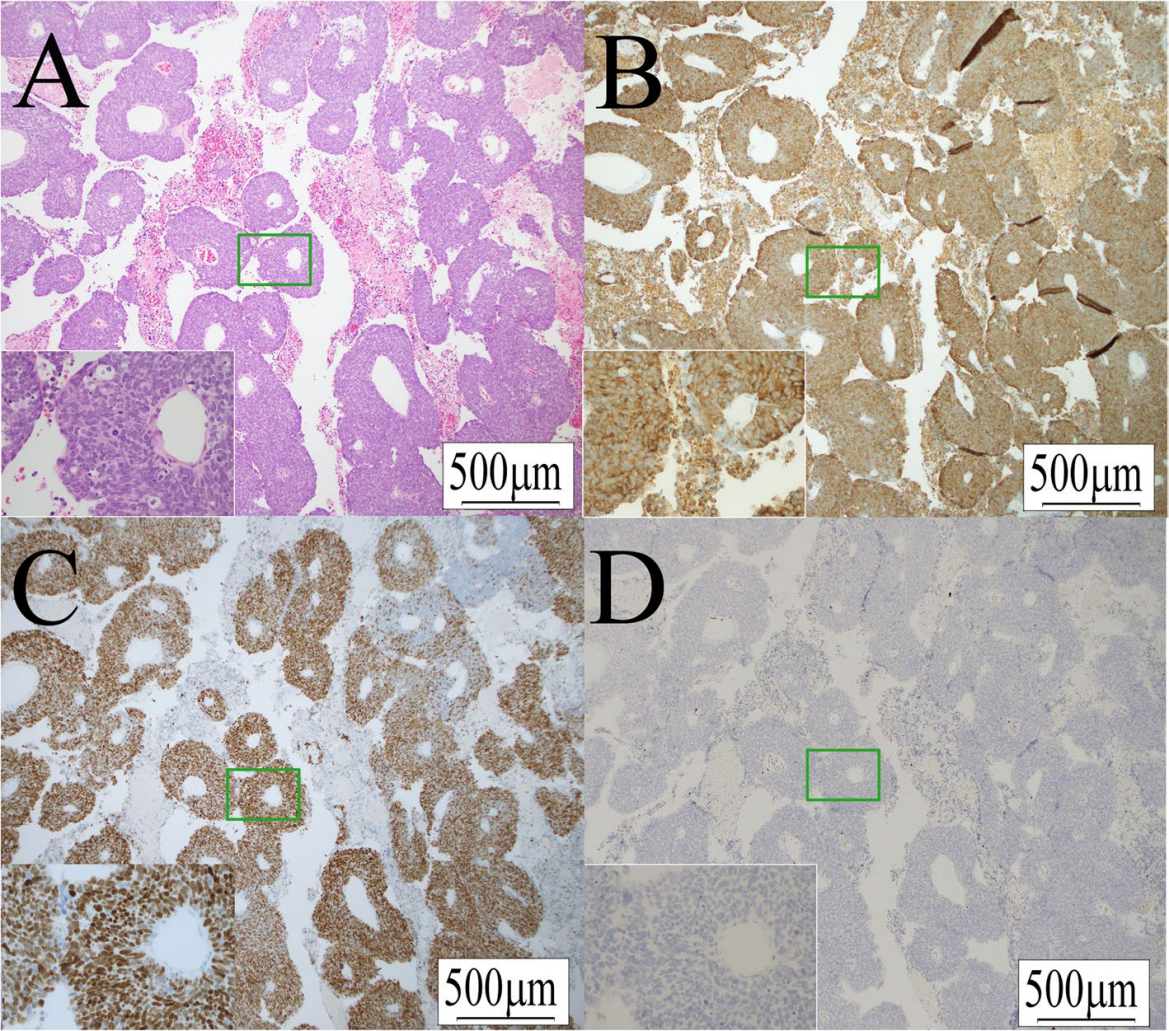
Histological images of small cell neuroendocrine carcinoma of the bladder with metastasis to the mediastinum (× 40). A magnified image (× 400) of the area in the green rectangle is shown in the bottom left. **A** Hematoxylin and eosin staining. Mitosis was noted in high power fields. **B** Synaptophysin. **C** GATA3. **D** CD5 shown by immunohistochemical staining

A histological examination including hematoxylin and eosin staining showed sheets of small cells with prominent nuclei, while mitoses and necrosis were also noted (Fig. 3A). Immunohistochemical staining results of markers specific for a neuroendocrine tumor (CD56, synaptophysin (Fig. 3B), chromogranin A) as well as GATA3 (Fig. 3C), specific for BC, were positive. On the other hand, CD5, a specific marker for primary thymic carcinoma, was negative (Fig. 3D). The same pathological results were noted for the primary BC, the diagnosis of which was revised to small cell carcinoma of bladder. Based on these findings, the right paracardiac mass was diagnosed as a metastatic small cell neuroendocrine carcinoma of the bladder.

The postoperative course was uneventful. However, 4 months after thoracic surgery, multiple areas of recurrence and metastasis were found in the mediastinum, liver, and pericardium in chest CT findings, and chemotherapy was started. At 10 months after thoracic surgery, each of the metastatic tumors had become reduced in size, though still remained, and follow-up examinations were continued.

## Discussion and conclusions

Previous reports of sites of mediastinal metastasis of a malignant tumor have noted the mediastinal lymph nodes [1], thymus [3], and pericardium [4, 5]. Thymic metastasis can be determined based on surgical or pathological findings. Metastasis to the mediastinal lymph nodes is diagnosed based on pathological findings showing the presence of a primary tumor [6], while that to the pericardium is diagnosed based on surgical findings, cytology findings of pericardial effusion, and/or pathological findings of a biopsy specimen taken from the pericardium [4, 5]. Additionally, metastatic tumors to the mediastinum related to various types of carcinomas have been reported to appear as an abnormal enlarged mass in chest CT images, with increased uptake noted in PET findings [7, 8]. In the present case, while the clinical history of BC and imaging findings for diagnosis suggested that the mediastinal tumor might be metastasis from BC, the malignant thymic epithelial tumor was considered to indicate a differential diagnosis for the isolated mediastinal tumor; thus, surgical resection was performed. As noted above, we were unable to precisely define the metastatic site based on pathological findings. However, intraoperative gross findings suggested that the tumor was located in pericardial fat tissue; thus, a diagnosis of mediastinal metastasis of BC was determined. The metastasis pathway in the present case is not clear. Common metastatic sites include the liver and lungs, as well as others in cases with a small cell carcinoma related to BC [2], indicating a hematogenous spread. Supradiaphragmatic lymph node metastasis from ovarian cancer has been reported [9]; thus, BC might share the same lymphatic route. Postoperative recurrence and findings of metastasis in the mediastinum, liver, and pericardium in the present case indicate that both lymphatic and hematogenous spreading are possible.

A small cell carcinoma of the bladder is very rare, comprising less than 1% of all malignant bladder tumors, and most often shows metastasis to regional lymph nodes, bone, liver, lung, or brain tissue, with other sites also seen [2]. Because of its rarity, the initial diagnosis of the primary tumor in the present case was urothelial carcinoma. Only a few cases of BC metastasis other than small cell carcinoma to mediastinal lymph nodes have been reported [10–12], and, to the best of our knowledge, there is no previous report of isolated mediastinal metastasis from a small cell carcinoma of the bladder. A small cell carcinoma related to BC is known for an aggressive clinical course, with early vascular and muscle invasion, and high propensity for metastasis; thus, the prognosis is poor, with an overall 5-year survival rate ranging from 8 to 25% [2]. In the present case, multiple metastasis lesions were noted at 4 months after chest surgery, even though the mediastinal tumor had been isolated based on preoperative PET imaging findings. Unfortunately, it was not possible to predict the poor prognosis of this patient because the small cell carcinoma was diagnosed after resection of mediastinal metastasis. With a primary aim of diagnosis as well as local disease control, surgical resection should be generally selected for isolated mediastinal metastasis from an extrathoracic carcinoma [12], as seen in the present case. Actually, the chemotherapeutic regimen for this patient was determined based on the pathological diagnosis of primary bladder cancer, which the mediastinal tumor provided an opportunity to re-evaluate. For surgical treatment of the condition, a less invasive thoracoscopic approach would have been preferable. However, an open thoracotomy was performed so as to avoid intraoperative injury to the large tumor, likely resulting in intrathoracic dissemination of cancer cells.

In conclusion, we performed surgery for mediastinal metastasis of bladder cancer, which was diagnosed as a small cell carcinoma developed from a metastatic tumor. Surgical resection for an isolated mediastinal tumor should be considered because its histological diagnosis can be useful for deciding appropriate therapeutic options.

## Data Availability

Not applicable.

## Abbreviations

BC: Bladder cancer
CT: Computed tomography
FDG: Fluorodeoxyglucose
PET: Positron emission tomography

## References

[1] Hess KR, Varadhachary GR, Taylor SH, Wei W, Raber MN, Lenzi R, et al. Metastatic patterns in adenocarcinoma. Cancer. 2006;106:1624–33. 16518827 10.1002/cncr.21778

[2] Al-Ahmadie H, Compérat E, Epstein JI. Neuroendocrine tumours. In: Moch H, Humphrey PA, Ulbright TM, Reuter VE, editors. WHO classification of tumours of urinary system & male genital organs. 4th ed. Lyon: IARC Press; 2016. p. 117–8.

[3] Omura A, Kimura K, Taniguchi S, Shintani Y. Thymic metastasis of ovarian cancer 33 years after primary surgery. Ann Thorac Surg. 2021;111:e361–3. 33130116 10.1016/j.athoracsur.2020.07.094

[4] Nasir N, Monroe CE, Hagerty BL, Quezado MM, Roth MJ, Schrump DS, et al. Adenoid cystic carcinoma of the salivary gland metastasizing to the pericardium and diaphragm: report of a rare case. Diagn Cytopathol. 2021;49:E31–5. 32770824 10.1002/dc.24566 PMC10763688

[5] Krywanczyk AR, Tan CD, Rodriguez ER. A clinico-pathologic approach to the differential diagnosis of pericardial tumors. Curr Cardiol Rep. 2021;23:119. 34269901 10.1007/s11886-021-01548-6

[6] Kubo N, Yoshizawa J, Hanaoka T. Solitary metastasis to a superior mediastinal lymph node after distal gastrectomy for gastric cancer: a case report. BMC Cancer. 2018;18:627. 29866101 10.1186/s12885-018-4555-7 PMC5987488

[7] Onal C, Findikcioglu A, Guler OC, Reyhan M. The use of 18F-FDG positron emission tomography to detect mediastinal lymph nodes in metastatic breast cancer. Breast. 2020;54:197–202. 33125983 10.1016/j.breast.2020.10.011 PMC7593617

[8] Lu P, Sun Y, Sun Y, Yu L. The role of 18F-FDG PET/CT for evaluation of metastatic mediastinal lymph nodes in patients with lung squamous-cell carcinoma or adenocarcinoma. Lung Cancer. 2014;85:53–8. 24792334 10.1016/j.lungcan.2014.04.004

[9] Hynninen J, Auranen A, Carpén O, Dean K, Seppänen M, Kemppainen J, et al. FDG PET/CT in staging of advanced epithelial ovarian cancer: frequency of supradiaphragmatic lymph node metastasis challenges the traditional pattern of disease spread. Gynecol Oncol. 2012;126:64–8. 22542580 10.1016/j.ygyno.2012.04.023

[10] Hiensch R, Belete H, Rashidfarokhi M, Galperin I, Shakil F, Epelbaum O. Unusual patterns of thoracic metastasis of urinary bladder carcinoma. J Clin Imaging Sci. 2017; 7:23. 10.4103/jcis.JCIS_9_17. 10.4103/jcis.JCIS_9_17 PMC5450457 28584690

[11] Iida K, Kawai N, Naiki T, Etani T, Ando R, Nagai T, et al. Case of metastatic urothelial carcinoma treated with pemetrexed as third-line chemotherapy with discussion and literature review. Case Rep Oncol. 2015;1(8):530–5. 10.1159/000442347 PMC4677708 26668577

[12] Riquet M, Berna P, Brian E, Badia A, Vlas C, Bagan P, et al. Intrathoracic lymph node metastases from extrathoracic carcinoma: the place for surgery. Ann Thorac Surg. 2009;88:200–5. 19559225 10.1016/j.athoracsur.2009.04.005

